# De-Escalated Adjuvant Radiation Therapy in Patients With HPV-Positive Oropharyngeal Cancer

**DOI:** 10.1001/jamanetworkopen.2026.12837

**Published:** 2026-05-18

**Authors:** Cecelia M. Hidalgo, Aaron W. Bogan, Katelyn S. Rourk, Hannah Q. Baratz, Daniel G. Eyassu, Katharine A. Price, Eric J. Moore, Samir H. Patel, Michael L. Hinni, Michelle A. Neben-Wittich, Yolanda I. Garces, Lisa A. McGee, Scott C. Lester, Jessica Wilson, Jean-Claude M. Rwigema, Ashish V. Chintakuntlawar, Homan Mohammadi, Adam L. Holtzman, Daniel L. Price, Jeffrey R. Janus, Joaquin J. Garcia, Robert L. Foote, Kendall K. Tasche, Daniel J. Ma, Kathryn M. Van Abel, Linda X. Yin, David M. Routman

**Affiliations:** 1 Department of Otolaryngology, Head and Neck Surgery, Mayo Clinic, Rochester, Minnesota; 2 Department of Quantitative Health Sciences, Division of Biostatistics, Mayo Clinic, Scottsdale, Arizona; 3 Division of Oncology, Mayo Clinic, Rochester, Minnesota; 4 Department of Radiation Oncology, Mayo Clinic, Phoenix, Arizona; 5 Department of Otolaryngology, Mayo Clinic, Phoenix, Arizona; 6 Department of Radiation Oncology, Mayo Clinic, Rochester, Minnesota; 7 Division of Medical Oncology, Mayo Clinic, Phoenix, Arizona; 8 Department of Radiation Oncology, Mayo Clinic, Jacksonville, Florida; 9 Department of Otolaryngology, Head and Neck Surgery, Mayo Clinic, Jacksonville, Florida; 10 Department of Laboratory Medicine & Pathology, Mayo Clinic, Rochester, Minnesota

## Abstract

**Importance:**

Standard adjuvant therapy for human papillomavirus–associated oropharyngeal squamous cell carcinoma (HPV-OPSCC) is radiation therapy 60 Gy with or without cisplatin delivered in 30 fractions during 6 weeks. A phase 3 randomized clinical trial demonstrated decreased toxic effects and improved patient-reported outcomes with de-escalated adjuvant radiation therapy (DART) compared with standard therapy; however, outcomes for patients treated in the clinical practice setting following trial adoption remain unknown.

**Objective:**

To compare characteristics and oncologic outcomes of patients treated with DART during clinical trials (on study) and in clinical practice settings (off study) and report progression-free survival (PFS) by risk category.

**Design, Setting, and Participants:**

This retrospective cohort study included patients treated with DART in the MC1273 and MC1675 trials and in clinical practice after study completion at a multisite tertiary care center. Patients with surgically resectable HPV-OPSCC who underwent margin-negative resection between August 20, 2013, and March 21, 2023, and were treated with DART were included in the analysis. Those with prior head and neck cancer, pT0 or pT4 disease, unknown HPV status, metastatic disease at presentation, synchronous primary tumors, induction chemotherapy, or treatment at an outside institution were excluded. Data were analyzed from July 1, 2024, to July 18, 2025.

**Interventions:**

DART consisting of 30 to 36 Gy in 1.5- to 1.8-Gy fractions twice daily with docetaxel 15 mg/m^2^ on days 1 and 8.

**Main Outcomes and Measures:**

Progression-free survival as measured via Kaplan-Meier analysis.

**Results:**

A total of 282 patients were included in the analysis: 176 (62.4%) in the on-study group and 106 (37.6%) in the off-study group (252 [89.4%] male; mean [SD] age, 59.1 [9.3] years). Both groups were predominantly male (157 [89.2%] and 95 [89.6%], respectively) and consisted mostly of never smokers (118 [67.0%] and 62 [58.5%], respectively). Pathologic features were similar between groups except for nodal stage (pN1: 155 [88.1%] on study vs 102 [96.2%] off study; pN2: 21 [11.9%] on study vs 4 [3.8%] off study; *P* = .02). The median follow-up times were 5.2 years (95% CI, 5.2-5.8 years) in the on-study group and 2.2 years (95% CI, 1.9-2.4 years) in the off-study group. Two-year PFS was similar (93% [95% CI, 88%-97%] on study vs 93% [95% CI, 88%-99%] off study). For patients with pN1 and no extranodal extension (ENE), 2- and 5-year PFS were 97% (95% CI, 93%-100%) and 96% (95% CI, 92%-100%), respectively. Patients with pN1 and ENE had a 2- and 5-year PFS of 93% (95% CI, 89%-98%) and 87% (95% CI, 81%-94%), respectively.

**Conclusions and Relevance:**

In this retrospective cohort study of patients receiving DART on and off study, oncologic outcomes were similar between the 2 groups. Fewer patients with pN2 received DART off study, given the interim results of MC1675. Patients with pN1 and no ENE treated with DART had excellent 2- and 5-year PFS, supporting DART as a compelling treatment option in well-selected patients. An additional study that is currently underway will evaluate biomarker risk-stratified treatment of patients with pN1 and ENE receiving DART.

## Key Points

**Question:**

Do patients who receive off-study de-escalated adjuvant radiation therapy (DART) have outcomes similar to those for patients who are treated during clinical studies?

**Findings:**

In this cohort study of 282 patients with human papillomavirus–associated oropharyngeal squamous cell carcinoma (HPV-OPSCC), 2-year outcomes were similar on and off study for local regional failure–free survival (97% vs 97%, respectively), progression-free survival (PFS [93% vs 93%, respectively]), and overall survival (98% vs 99%, respectively). For patients at intermediate risk, 2- and 5-year PFS were 97% and 96%, respectively; for those at high risk, 2- and 5-year PFS were 93% and 87%, respectively.

**Meaning:**

These findings suggest that patients with HPV-OPSCC who are treated with DART have excellent oncologic outcomes, especially patients with intermediate-risk disease.

## Teaser

This cohort study explores the characteristics and oncologic outcomes of patients with human papillomavirus (HPV)–associated oropharyngeal squamous cell carcinoma who were treated with de-escalated adjuvant radiation therapy and assesses progression-free survival by risk category.

## Introduction

Treatment de-escalation for human papillomavirus–associated oropharyngeal squamous cell carcinoma (HPV-OPSCC) has been studied, given the toxic effects associated with standard therapy as well as the improved prognosis compared with other HPV-negative squamous cell carcinomas of the head and neck.^1^ MC1273 was a phase 2 study investigating de-escalated adjuvant radiation therapy (DART) after margin-negative resection.^2^ Two randomized studies, including the follow-up phase 3 trial MC1675 and ECOG 3311, demonstrated excellent overall outcomes, including high rates of local regional tumor control with adjuvant de-escalated therapy.^3,4,5^ Additionally, treatment de-escalation resulted in decreased clinician-assessed and patient-reported toxic effects in MC1273 and MC1675 as well as reductions in global costs compared with standard-of-care adjuvant radiation therapy.^4,6,7^

Given the clinically meaningful improvements in swallow function, speech, sexual function, socialization, and dryness of mouth seen with de-escalation compared with standard therapy along with the excellent rates of cancer control, DART was adopted as a standard of care at our institution prior to the ongoing follow-up study DART 2.^4^ This period of de-escalation therapy in clinical practice settings (hereinafter referred to as off study) presented an opportunity to evaluate the outcomes of patients treated outside a clinical trial. Based on extensive phase 4 drug literature, effectiveness in standard clinical practice often struggles to meet the outcomes of highly select populations treated during the study (hereinafter referred to as on study).^8,9^

Herein, we analyzed characteristics and outcomes of patients treated with DART during a phase 2 study (MC1273), a phase 3 study (MC1675), and in phase 4 (off study). We sought to understand whether patients treated off study in a standard clinical practice setting have the same rates of cancer control seen on study and to assess the overall effectiveness of DART in this large patient cohort treated during the last decade.

## Methods

### Study Design

Institutional review board approval from the Mayo Clinic was obtained for this retrospective cohort study, and all patients who had not provided written informed consent to participate in retrospective research were removed in accordance with institutional review board requirements to ensure patient privacy and ethical data use. Patients with HPV-OPSCC who received de-escalated adjuvant therapy as part of a prospective clinical trial were compared with those who received the same treatment off study. While the on-study group was prospectively treated per protocol (MC1273 and MC1675), the off-study group received similar de-escalated adjuvant therapy at the discretion of the treating clinicians. The DART regimen is an aggressive de-escalation of adjuvant therapy in decreased radiation dose and in the radiosensitizer used. The regimen consists of 30 to 36 Gy in 1 × 5- to 1 × 8-Gy fractions twice per day for 2 weeks plus intravenous docetaxel 15 mg/m^2^ on days 1 and 8 of treatment. This study conducted ad hoc, observational analyses independent of the prospective trial protocols. All data were retrospectively collected, analyzed, and reported using the Strengthening the Reporting of Observational Studies in Epidemiology (STROBE) reporting guideline for cohort studies.

### Patient Selection

Eligible patients underwent surgical resection of HPV-OPSCC from August 20, 2013, to March 21, 2023. HPV positivity was defined by p16 immunohistochemistry, HPV DNA in situ hybridization, or HPV RNA in situ hybridization. All patients had negative surgical margins confirmed on final pathologic evaluation. Patients were excluded if they had a history of head and neck cancer, had pT0 or pT4 disease based on American Joint Committee on Cancer (AJCC) eighth edition staging, or had received induction chemotherapy. Additional exclusion criteria included presence of a synchronous oropharyngeal primary tumor, unknown HPV status, and failure to complete the prescribed treatment regimen.

### Data Collection

Characteristics of study participants, including age, sex, and smoking history, were collected. Area Deprivation Index (ADI) national percentile ranks were obtained from the Neighborhood Atlas (University of Wisconsin School of Medicine and Public Health, Madison) and linked to patient data via census block group using 5-digit zip codes. Patient-level ADI values were derived by calculating the mean block group ADI scores for zip codes spanning multiple block groups. Pathologic risk factors, including extranodal extension (ENE), lymphovascular invasion (LVI), perineural invasion (PNI), number of lymph nodes involved, and size of the largest node involved, were also collected. The AJCC eighth edition staging system was used for this analysis. Adjuvant treatment information was collected, including radiation treatment start and end dates, total dose (in Gy) and total fractions delivered, and chemotherapy agent given. Follow-up, recurrence data, and oncologic outcome variables were collected to the last record update for each patient. Typically, patients were followed up every 3 to 4 months for the first 2 years, every 6 months for years 3 to 5, and yearly thereafter beginning at the end of radiation treatment. This was adjusted if patients had complications or personal constraints. All data were manually abstracted from the electronic medical record by trained researchers from November 22, 2013, to July 24, 2024, and stored in a Research Data Capture (REDCap) database specifically designed for ongoing data collection. Five analyzed variables were missing data after abstraction. These included oropharyngeal primary disease site (10 of 282 [3.5%]), PNI (2 of 282 [0.7%]), LVI (2 of 282 [0.7%]), pathologic tumor size (13 of 282 [4.6%]), and size of largest involved node (8 of 282 [2.8%]). Because the proportion of missing data for each variable was small, analyses involving these variables were performed using complete case analysis, with any cases containing missing values excluded from the respective analyses.

### Statistical Analysis

Data were analyzed from July 1, 2024, to July 18, 2025. Categorical variables were compared using Pearson χ^2^ test, and continuous variables were compared using the Kruskal-Wallis rank sum test or linear model analysis of variance. Shapiro-Wilk tests for normality were performed on continuous variables to validate parametric testing assumptions. Kaplan-Meier analysis and restricted mean survival time were used to compare survival outcomes, including overall survival (OS), disease-specific survival (DSS), progression-free survival (PFS), and locoregional failure–free survival (LRFFS). Restricted mean survival time was calculated at 2 years. All survival times were calculated from the date of surgery. Univariable Cox proportional hazards regression models were fit to evaluate whether observed associations among patient, treatment, and diagnostic characteristics and oncologic outcomes were consistent with those previously reported in the literature. The results are detailed in the eFigure in Supplement 1. Multivariable Cox proportional hazards regression models were fit to examine associations between oncologic outcomes and de-escalation in the on-study and off-study groups when adjusted for age at surgery and disease severity as defined by AJCC eighth edition overall stage. Two-sided *P* < .05 was considered statistically significant. All CIs were calculated at the 95% level unless explicitly stated otherwise. All statistical analysis was performed using R, version 4.4.1, in RStudio (R Project for Statistical Computing) by a senior statistician (A.W.B.).

## Results

### Characteristics of Study Participants

**Figure 1.**
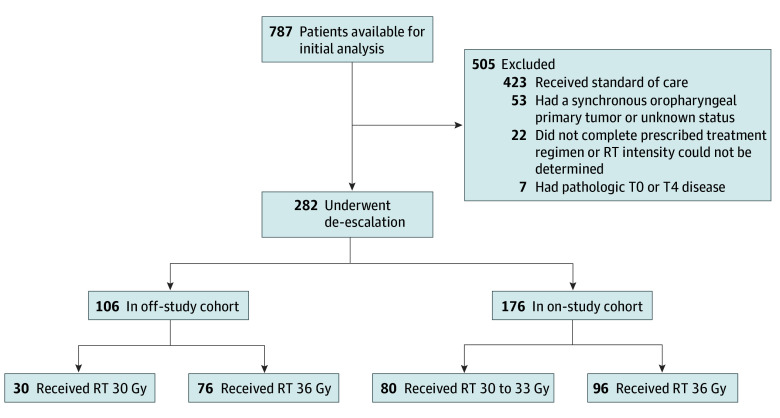
CONSORT Diagram All radiation therapy (RT) was delivered in 20 fractions.

Of the 282 patients included for analysis, 176 (62.4%) were treated on study and 106 (37.6%) were treated off study (Figure 1). Seventeen patients (6.0%) were treated at Mayo Clinic Health System locations in Rochester, Minnesota, or Eau Claire and Lacrosse, Wisconsin; 20 patients (7.1%) were treated at the Mayo Clinic’s Phoenix, Arizona, campus; and 245 patients (86.9%) were treated at Mayo Clinic, Rochester. The overall cohort was predominantly male (252 [89.4%] compared with 30 [10.6%] female), with a mean (SD) age of 59.1 (9.3) years at the time of surgery, and most were never smokers (180 [63.8%]). The median ADI in the sample was 49.6 (IQR, 34.2-61.9), demonstrating a range of socioeconomic disadvantage that approximates the national distribution of ADI. The primary oropharyngeal subsite was the tonsil (148 of 272 [54.4%]), followed by the base of the tongue (124 of 272 [45.6%]), with 10 cases that could not be determined. The pathologic tumor stage (pT), size of the largest involved lymph node, and presence of PNI, LVI, and ENE were similar between groups (Table 1). However, pathologic nodal stage differed, with more patients with pN2 receiving de-escalated treatment on study (21 of 176 [11.9%]) than off study (4 of 106 [3.8%]). This was expected based on practice changes implemented in response to the initial results of MC1675.^5^ Also as expected, earlier treatment dates for most patients in the on-study group (median surgery date, July 10, 2017, vs March 30, 2022) resulted in longer median follow-up time for patients in the on-study group vs those in the off-study group. Reverse Kaplan-Meier median follow-up for the on-study group was 5.2 (95% CI, 5.2-5.8) years vs 2.2 (95% CI, 1.9-2.4) years for the off-study group. Because of this limitation, we limited the analysis to 2-year outcomes for the off-study group.

**Table 1.** Patient Characteristics

Characteristic	Treatment group, No. (%)			*P* value
	Off study (n = 106)	On study (n = 176)^a^	Total (N = 282)	
Age at surgery, mean (SD), y	60.0 (9.4)	58.5 (9.2)	59.1 (9.3)	.20^b^
Sex				
Female	11 (10.4)	19 (10.8)	30 (10.6)	.91^c^
Male	95 (89.6)	157 (89.2)	252 (89.4)	
Tobacco smoking				
Current	6 (5.7)	3 (1.7)	9 (3.2)	.11^c^
Never	62 (58.5	118 (67.0)	180 (63.8)	
Past	38 (35.8)	55 (31.3)	93 (33.0)	
Primary oropharyngeal site^d^				
Base of tongue	45 (46.4)	79 (45.1)	124 (45.6)	.84^c^
Tonsil	52 (53.6)	96 (54.9)	148 (54.4)	
Pathologic tumor classification				
pT1	50 (47.2)	71 (40.3)	121 (42.9)	.44^c^
pT2	48 (45.3)	86 (48.9)	134 (47.5)	
pT3	8 (7.5)	19 (10.8)	27 (9.6)	
Pathologic nodal classification				
pN1	102 (96.2)	155 (88.1)	257 (91.1)	.02^c^
pN2	4 (3.8)	21 (11.9)	25 (8.9)	
Oropharyngeal tumor pathologic size, mean (SD), cm^e^	2.2 (1.3)	2.4 (1.2)	2.3 (1.2)	.18^b^
Total No. of positive nodes, mean (SD)	2.2 (2.9)	2.5 (2.4)	2.4 (2.6)	.35^b^
Size of largest node involved by tumor, mean (SD), cm^f^	3.9 (1.3)	3.9 (1.3)	3.9 (1.3)	.64^b^
Extranodal extension				
No	50 (47.2)	78 (44.3)	128 (45.4)	.64^c^
Yes	56 (52.8)	98 (55.7)	154 (54.6)	
Perineural invasion^g^				
No	86 (81.1)	139 (79.9)	225 (80.4)	.54^c^
Yes	20 (18.9)	33 (19.0)	53 (18.9)	
Indeterminate	0	2 (1.1)	2 (0.7)	
Lymphovascular invasion^g^				
No	85 (80.2)	131 (75.3)	216 (77.1)	.17^c^
Yes	20 (18.9)	34 (19.5)	54 (19.3)	
Indeterminate	1 (0.9)	9 (5.2)	10 (3.6)	

^a^ Includes 112 patients from the MC1675 study and 64 from the MC1273 study.

^b^ Calculated using linear model analysis of variance.

^c^ Calculated using the Pearson χ^2^ test.

^d^ Includes 272 patients in whom site could be determined.

^e^ Includes 269 patients.

^f^ Includes 274 patients.

^g^ Includes 280 patients.

### Survival Outcomes in Overall Cohort

**Figure 2.**
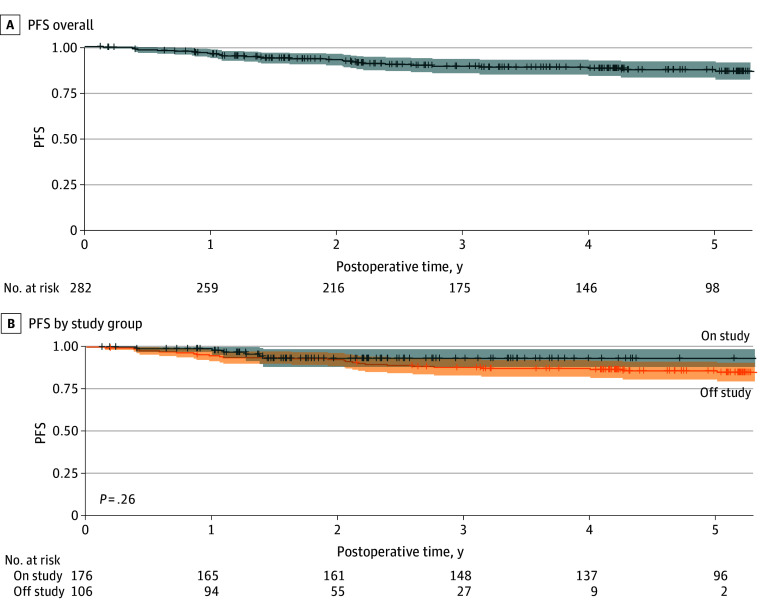
Progression-Free Survival (PFS) for Patients Receiving De-Escalated Adjuvant Radiation Therapy Overall and by Study Status The median PFS follow-up was 5.2 years (95% CI, 5.2-5.8 years) for patients in the on-study group and 2.2 years (95% CI, 1.9-2.4 years) for patients in the off-study group.

For all patients combined (on and off study), the 2-year PFS was 93% (95% CI, 90%-96%) and the 5-year PFS was 87% (95% CI, 83%-92%) (Figure 2A). The 2-year OS was 98% (95% CI, 96%-100%) and the 5-year OS was 94% (95% CI, 91%-98%). The 2-year LRFFS was 97% (95% CI, 95%-99%) and the 5-year LRFFS was 95% (95% CI, 93%-98%). DSS at 2 years was 99% (95% CI, 98%-100%) and at 5 years was 96% (95% CI, 94%-99%) (eTable 1 in Supplement 1).

### Survival Outcomes by Study Status

PFS outcomes and RM-PFS at 2 years were similar between the on-study and off-study groups. The 2-year PFS for the on-study group was 93% (95% CI, 88%-97%) and the 5-year PFS was 86% (95% CI, 81%-91%) compared with a 2-year-PFS of 93% (95% CI, 88%-99%) for the off-study group (*P* = .26) (Figure 2B); the difference in the restricted mean PFS at 2 years was 0.03 (95% CI, −0.04 to 0.10) (*P* = .45). The 2-year OS for the on-study group was 98% (95% CI, 96%-100%) and the 5-year OS was 94% (95% CI, 90%-98%) compared with a 2-year OS of 99% (95% CI, 97%-100%) for the off-study group (*P* = .63); the restricted mean OS difference at 2 years was 0.01 (95% CI, −0.02 to 0.04) (*P* = .43). The 2-year LRFFS for the on-study group was 97% (95% CI, 95%-100%) and the 5-year LRFFS was 95% (95% CI, 92%-99%) compared with a 2-year LRFFS of 97% (95% CI, 93%-100%) for the off-study group (*P* = .82) (Table 2); the restricted mean LRFFS difference at 2 years was 0.003 (95% CI, −0.04 to 0.05) (*P* = .91). There were too few observations to report 5-year outcomes for patients in the off-study group (number at risk: 2 of 106 at 5 years).

**Table 2.** Survival Outcomes by Study Status

Outcome	Treatment group, % (95% CI)		*P* value
	On study	Off study^a^	
PFS			
2 y	93% (88-97)	93% (88-99)	.26
5 y	86% (81-91)	NA	
OS			
2 y	98% (96-100)	99% (97-100)	.63
5 y	94% (90-98)	NA	
LRFFS			
2 y	97% (95-100)	97% (93-100)	.82
5 y	95% (92-99)	NA	

Abbreviations: LRFFS, locoregional recurrence failure–free survival; NA, not applicable; OS, overall survival; PFS, progression-free survival.

^a^ There were too few observations to report 5-year outcomes for patients in the off-study groups (number at risk: 2 of 106 at 5 years).

Multivariable Cox proportional hazards regression models for OS, DSS, PFS, and LRFFS included age at surgery, AJCC eighth edition overall stage (I vs II and III), and study status. Stages II and III were combined due to the small number of patients with stage III cancer (4 of 282 [1.4%]). Study status was not associated with any oncologic outcomes (eTable 2 in Supplement 1).

### Overall Cohort: ENE and pN1

**Figure 3.**
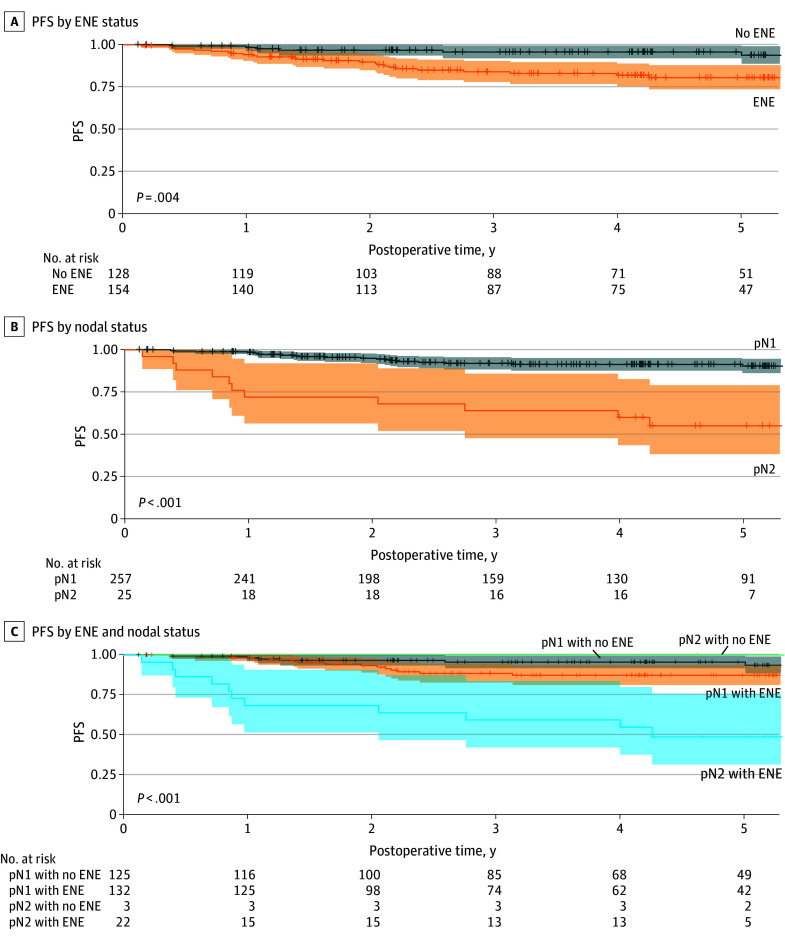
Progression-Free Survival (PFS) for Patients With T1 to T3 Disease Patients were stratified by extranodal extension (ENE) status, pathologic nodal stage (pN1 or pN2), and all combinations of pathologic nodal stage and ENE status.

PFS for patients with no ENE was 97% (95% CI, 94%-100%) at 2 years and 96% (95% CI, 92%-100%) at 5 years compared with 90% (95% CI, 85%-95%) at 2 years and 80% (95% CI, 74%-88%) at 5 years for patients with ENE (*P* = .004) (Figure 3A). PFS for patients with pN1 was 95% (95% CI, 92%-98%) at 2 years and 91% (95% CI, 88%-95%) at 5 years compared with 72% (95% CI, 56%-92%) at 2 years and 55% (95% CI, 38%-79%) at 5 years for patients with pN2 (*P* < .001) (Figure 3B). PFS for patients with pN1 and no ENE (intermediate risk) was 97% (95% CI, 93%-100%) at 2 years and 96% (95% CI, 92%-100%) at 5 years. For patients with pN1 and ENE (high risk), PFS was 93% (95% CI, 89%-98%) at 2 years and 87% (95% CI, 81%-94%) at 5 years (Figure 3C). Due to differences in follow-up time between groups, combined 5-year survival outcomes largely reflect the experiences of patients in the on-study group.

## Discussion

Criteria for the adoption of treatment de-escalation for HPV-OPSCC have remained a matter of debate. The NRG-HN005 study^10^ provided compelling evidence that de-escalated primary chemoradiation results in inferior PFS compared with high-intensity therapy. Patient selection is likely critical to appropriate de-escalation. Adjuvant de-escalation allows for more informed patient selection based on the results of surgery and pathologic factors as demonstrated by MC1675 (DART)^4^ and ECOG 3311.^3^ However, additional off-study data are lacking. Evaluations or standard clinical practice outcomes for observational, noninterventional trials in a clinical setting following randomized clinical trials are necessary. These reports are common in the postmarketing surveillance of drugs but less common for radiation therapy regimens. In the present study, we investigated the pooled outcomes of 282 patients treated with DART during 2 prospective trials and after study completion in routine care. Based on the initial results of MC1675,^5^ de-escalation for patients with pN2 (≥5 nodes involved) was used selectively off study in only 4% of patients. Otherwise, the populations in the on- and off-study groups were similar, as were the outcomes, with the remaining discussion focused on patients with pN1.

For patients at intermediate risk patients (with pT1-pT3 pN1 disease without ENE), PFS was 97% at 2 years and 96% at 5 years. For comparison, intermediate-risk patients receiving the 60-Gy standard of care treatment in MC1675 and ECOG 3311 had numerically lower 2-year PFS of 95% to 96% in each trial.^3,4^ Given these high rates of PFS in the phase 2, phase 3, and phase 4 settings, it is highly unlikely that standard adjuvant therapy would offer any benefit in cancer treatment, especially considering the DART regimen demonstrated less clinician-assessed toxic effects as well as improved patient reported outcomes.^4,6^ These data support the use of DART as a new treatment option supported by level 1 evidence and retrospective analysis as presented herein for intermediate-risk patients with pT1 to pT3 pN1 disease (no ENE) meeting criteria.

For patients at high risk, as defined by ENE (pT1-pT3 pN1) and representing approximately 50% of the population, 2- and 5-year PFS was 93% and 87%, respectively. The predominant pattern of disease progression is distant, with most events in the first year after treatment, suggesting early micrometastatic disease giving rise to recurrence. Additionally, the effect is most pronounced in patients with pN2. Whether or not increased rates of recurrence for high-risk patients with ENE and pN2 is due to minimal exposure to systemic chemotherapy with the use of low-dose docetaxel or another phenomenon is unknown.

Further work is needed to improve patient selection in the high-risk cohort with pN1 and ENE. ECOG 3311 used the extent of ENE of less than 1 mm as a criterion, with a total of 18 patients with ENE receiving de-escalation.^3^ Nodal count is another promising metric, where patients with 1 to 2 nodes and ENE may be at lower risk than patients with 3 to 4 nodes and ENE.^11^ A secondary analysis of MC1675 demonstrated minimal residual disease status based on circulating tumor HPV DNA (ctHPVDNA) could inform patient selection.^12^ Additional studies have demonstrated tumor immune cell infiltrate can effectively stratify patients.^13,14^ Nevertheless, whether ENE extent, nodal count, ctHPVDNA, genetic signatures, or immune signatures are used to refine the population with pN1 and ENE suitable for de-escalation, noninferiority trials are not feasible using each criterion.^15^ Patients at our institution with ENE generally receive de-escalation on study only (DART 2), using ctHPVDNA in combination with pathologic risk factors to refine patient selection for de-escalation. Informed discussion of the trade-offs of DART and standard of care is needed, given that risk and treatment intensity are a continuum. Discrete choice experiments may be particularly useful in this instance to inform future decision-making.^16^

### Limitations

Although this study represents a large analysis of patients treated during more than a decade across centers in 3 states, it has limitations that may impact generalizability of the results. These include but are not limited to standardized practice at tertiary care centers with intraoperative frozen section margin assessment in a relatively homogenous patient population. Most of the patients were treated at Mayo Clinic, Rochester, in a setting that is well resourced and with a weekly multidisciplinary clinic dedicated to this disease process. Further external validation is warranted to help substantiate generalizability. Additionally, extent of ENE was not quantified or used as a selection criterion, where ECOG 3311 used 1 mm as a threshold^3^ and the American Society for Clinical Oncology transoral robotic surgery guidelines recommend de-escalated therapy for patients with less than 1 mm of ENE.^17^ There were only 3 patients with pN2 and no ENE in the cohort, which substantially limits any inference concerning DART’s efficacy for these patients. Finally, twice-daily fractionation in the DART regimen has not been widely adopted. It is unknown whether 30 to 36 Gy delivered at 2 Gy daily would have equivalent toxicity profiles and outcomes. Twice-daily treatment may be a barrier to implementation of the DART regimen, depending on patient and treatment center factors. Once-daily treatment may be more convenient and practical depending on practice setting.

As with all retrospective cohort studies, latent confounding limits causal inference, particularly given the inclusion of both on-study and off-study patient groups. Although multivariable adjustments were applied for known or suspected confounders, residual confounding cannot be excluded. Additionally, the use of retrospectively collected clinical data introduces the potential for data recording errors, misclassification, and missing information, which may influence the results. Patients treated in this study spanned a decade of clinical practice, and the gold standard for HPV testing changed during the study period. As such, p16 positivity was used as the definition of HPV positivity rather than the current gold standard of E6/7 RNA in situ hybridization. Given the rate of 98% concordance in North America between HPV and p16 positivity and that outcomes were favorable, p16 positivity as a surrogate for HPV positivity was highly unlikely to have any impact on outcomes, but it remains a limitation.

As expected, earlier treatment dates among patients in the on-study group resulted in longer median follow-up compared with the off-study group (5.2 vs 2.2 years). Given this imbalance, conclusions for the off-study group were restricted to 2-year outcomes. Accordingly, any 5-year inferences derived from these results primarily reflect the experience of patients undergoing on-study de-escalation.

## Conclusions

In this retrospective cohort study of 282 patients treated on and off study, DART was associated with excellent oncologic outcomes in addition to the previously demonstrated benefits in toxic effects. For patients with pT1 to pT3 and N1 without ENE, DART represents a new treatment option for well-selected patients, supported by level 1 evidence and the retrospective analysis presented herein.

## Notes

Supplement 1. **eTable 1.** Survival Outcomes in Overall Cohort (On-Study and Off-Study Groups) **eTable 2.** Multivariable Cox Proportional Hazards Regression Model Summaries **eFigure.** Univariable Descriptive Analysis: Cox Proportional Hazards Regression Models

Supplement 2.
Data Sharing Statement
