# Supplementary material for: De-Escalated Adjuvant Radiation Therapy in Patients With HPV-Positive Oropharyngeal Cancer
Source: JAMA Netw Open. 2026 May 18;9(5):e2612837. doi: 10.1001/jamanetworkopen.2026.12837 (PMC13184782; doi:10.1001/jamanetworkopen.2026.12837)
Supplement: Supplement 1. — eTable 1. Survival Outcomes in Overall Cohort (On-Study and Off-Study Groups) eTable 2. Multivariable Cox Proportional Hazards Regression Model Summaries eFigure. Univariable Descriptive Analysis: Cox Proportional Hazards Regression Models [file jamanetwopen-e2612837-s001.pdf]

Supplementary Online Content

Hidalgo CM, Bogan AW, Rourk KS, et al. De-escalated adjuvant radiation therapy for patients with HPV-positive oropharyngeal cancer. *JAMA Netw Open.* 2026;9(5):e2612837. doi:10.1001/jamanetworkopen.2026.12837

eTable 1. Survival Outcomes in Overall Cohort (On-Study and Off-Study Groups)

Table 2. Multivariable Cox Proportional Hazards Regression Model Summaries

eFigure. Univariable Descriptive Analysis: Cox Proportional Hazards Regression Models

This supplementary material has been provided by the authors to give readers additional information about their work.

© 2026 Hidalgo CM et al. *JAMA Network Open*.

eTable 1. Survival Outcomes in Overall Cohort (On-Study and Off-Study Groups)

Time Point	PFS (95%CI)	OS (95%CI)	LRFFS (95%CI)	DSS (95%CI)	DMFS (95%CI)
**2-year**	93% (90–96%)	98% (96–100%)	97% (95–99%)	99% (98–100%)	98% (96–100%)
**5-year**	87% (83–92%)	94% (91–98%)	95% (93–98%)	96% (94–99%)	94% (91–98%)

PFS: Progression-free survival, OS: Overall survival, LRFFS: Locoregional recurrence failure free survival, DSS: Disease specific survival, DMFS: Distant metastasis-free survival

eTable 2. Multivariable Cox Proportional Hazards Regression Model Summaries

a)

OS (n = 282, 5.0% event rate). Age modeled as time dependent.

	Regression Coefficients		Adjusted Hazard Ratio		
**Characteristic**	**log(HR)**	**95% CI**	**HR**	**95% CI**	**p-value**
**Age at Surgery**	0.15	0.03, 0.26	1.16	1.03, 1.30	0.01
**Age at Surgery Time Correction**	-0.04	-0.07, -0.01	0.96	0.93, 0.99	0.008
**AJCC 8 Stage**					0.02
Stage I	—	—	—	—	
Stage II/III	1.24	0.16, 2.32	3.45	1.17, 10.14	0.02
**Study Status**					0.52
Off Study	—	—	—	—	
On Study	0.52	-1.06, 2.09	1.68	0.35, 8.07	0.52

Abbreviations: CI = Confidence Interval, HR = Hazard Ratio, OS = Overall Survival

© 2026 Hidalgo CM et al. *JAMA Network Open*.

b)

DSS (n = 282, 2.8% event rate). Age modeled as time dependent.

	Regression Coefficients		Adjusted Hazard Ratio		
**Characteristic**	**log(HR)**	**95% CI**	**HR**	**95% CI**	**p-value**
**Age at Surgery**	0.19	0.01, 0.37	1.21	1.01, 1.44	0.04
**Age at Surgery Time Correction**	-0.06	-0.10, -0.01	0.95	0.91, 0.99	0.01
**AJCC 8 Stage**					0.03
Stage I	—	—	—	—	
Stage II/III	1.61	0.17, 3.06	5.02	1.18, 21.33	0.03
**Study Status**					0.64
Off Study	—	—	—	—	
On Study	0.53	-1.66, 2.73	1.70	0.19, 15.30	0.64

Abbreviations: CI = Confidence Interval, HR = Hazard Ratio, DSS = Disease Specific Survival

© 2026 Hidalgo CM et al. *JAMA Network Open*.

c)

PFS (n = 282, 11.3% event rate). Age modeled as time dependent.

	Regression Coefficients		Adjusted Hazard Ratio		
**Characteristic**	**log(HR)**	**95% CI**	**HR**	**95% CI**	**p-value**
**Age at Surgery**	0.08	0.02, 0.15	1.09	1.02, 1.16	0.01
**Age at Surgery Time Correction**	-0.03	-0.05, 0.00	0.98	0.95, 1.00	0.06
**AJCC 8 Stage**					0.006
Stage I	—	—	—	—	
Stage II/III	1.01	0.29, 1.73	2.74	1.33, 5.62	0.006
**Study Status**					0.22
Off Study	—	—	—	—	
On Study	0.57	-0.35, 1.48	1.76	0.71, 4.39	0.22

Abbreviations: CI = Confidence Interval, HR = Hazard Ratio, PFS = Progression Free Survival

© 2026 Hidalgo CM et al. *JAMA Network Open*.

d)

LRFFS (n = 282, 4.3% event rate). Age modeled as time dependent.

	Regression Coefficients		Adjusted Hazard Ratio		
**Characteristic**	**log(HR)**	**95% CI**	**HR**	**95% CI**	**p-value**
**Age at Surgery**	0.11	0.01, 0.21	1.12	1.01, 1.24	0.04
**Age at Surgery Time Correction**	-0.04	-0.08, 0.00	0.96	0.92, 1.00	0.04
**AJCC 8 Stage**					0.18
Stage I	—	—	—	—	
Stage II/III	0.83	-0.38, 2.04	2.29	0.68, 7.69	0.18
**Study Status**					0.74
Off Study	—	—	—	—	
On Study	0.23	-1.13, 1.59	1.26	0.32, 4.92	0.74

Abbreviations: CI = Confidence Interval, HR = Hazard Ratio, LFRRS = Locoregional Failure Free Survival

© 2026 Hidalgo CM et al. *JAMA Network Open*.

eFigure. Univariable Descriptive Analysis: Cox Proportional Hazards Regression Models

Univariable Cox proportional hazards regressions were fit to assess the consistency of observed associations between patient, treatment, and diagnostic characteristics and oncologic outcomes with those reported in the literature. Because these analyses were intended to be descriptive rather than inferential, no correction for multiple comparisons was applied, and p-values are presented for descriptive purposes only.

a) OS

Univariable Cox Proportional Hazards Models

Variable		N	Hazard ratio	p
**Study Status**	Off Study	106	Reference	
	On Study	176	1.45 (0.31, 6.86)	0.64
**Age at Surgery**		282	1.02 (0.97, 1.09)	0.41
**Sex**	Female	30	Reference	
	Male	252	1.60 (0.21, 12.20)	0.65
**Pack Years**		278	1.02 (0.99, 1.06)	0.17
**Primary Tumor Site**	Base of Tongue	124	Reference	
	Tonsil	148	0.49 (0.16, 1.47)	0.20
**Primary Tumor Size (cm)**		269	1.34 (0.87, 2.08)	0.19
**pT**	T1	121	Reference	
	T2	134	2.81 (0.76, 10.37)	0.12
	T3	27	3.05 (0.51, 18.29)	0.22
**Number of Involved Nodes**		282	1.10 (1.00, 1.21)	0.06
**pN**	pN1	257	Reference	
	pN2	25	5.23 (1.75, 15.65)	0.003
**Extranodal Extension**	No	128	Reference	
	Yes	154	3.19 (0.89, 11.44)	0.08
**Perineural Invasion**	No	227	Reference	
	Yes	53	0.71 (0.16, 3.18)	0.66
**Lymphovascular Invasion**	No	226	Reference	
	Yes	54	1.80 (0.56, 5.74)	0.32
**Total Dose**		282	1.19 (0.96, 1.47)	0.11

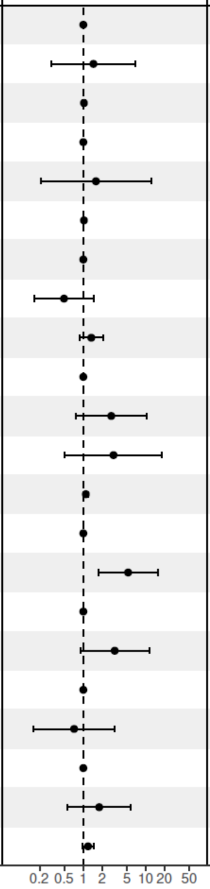

© 2026 Hidalgo CM et al. *JAMA Network Open*.

b) DSS

Univariable Cox Proportional Hazards Models

Variable		N	Hazard ratio		p
**Study Status**	Off Study	106		Reference	
	On Study	176		1.42 (0.16, 12.50)	0.75
**Age at Surgery**		282		1.00 (0.93, 1.08)	0.93
**Sex**	Female	30		Reference	
	Male	252		0.88 (0.11, 7.17)	0.91
**Pack Years**		278		1.03 (0.98, 1.07)	0.23
**Primary Tumor Site**	Base of Tongue	124		Reference	
	Tonsil	148		0.53 (0.13, 2.21)	0.38
**Primary Tumor Size (cm)**		269		1.34 (0.76, 2.39)	0.31
**pT**	T1	121		Reference	
	T2/T3	161		5.37 (0.66, 43.65)	0.12
**Number of Involved Nodes**		282		1.13 (1.02, 1.25)	0.02
**pN**	pN1	257		Reference	
	pN2	25		9.20 (2.29, 36.92)	0.002
**Extranodal Extension**	No	128		Reference	
	Yes	154		6.12 (0.75, 49.76)	0.09
**Perineural Invasion**	No	227		Reference	
	Yes	53		1.42 (0.29, 7.03)	0.67
**Lymphovascular Invasion**	No	226		Reference	
	Yes	54		1.48 (0.30, 7.36)	0.63
**Total Dose**		282		1.33 (0.94, 1.89)	0.11

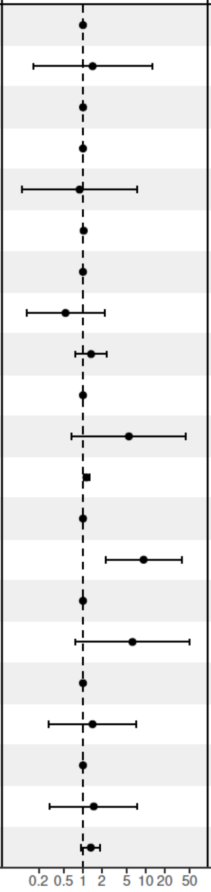

© 2026 Hidalgo CM et al. *JAMA Network Open*.

c) PFS

Univariable Cox Proportional Hazards Models

Variable		N	Hazard ratio		p
**Study Status**	Off Study	106		Reference	
	On Study	176		1.67 (0.67, 4.14)	0.27
**Age at Surgery**		282		1.04 (1.00, 1.08)	0.06
**Sex**	Female	30		Reference	
	Male	252		1.88 (0.45, 7.86)	0.39
**Pack Years**		278		1.02 (1.00, 1.05)	0.05
**Primary Tumor Site**	Base of Tongue	124		Reference	
	Tonsil	148		0.98 (0.49, 1.96)	0.95
**Primary Tumor Size (cm)**		269		1.13 (0.84, 1.51)	0.41
**pT**	T1	121		Reference	
	T2	134		1.92 (0.90, 4.11)	0.09
	T3	27		0.85 (0.19, 3.86)	0.83
**Number of Involved Nodes**		282		1.10 (1.04, 1.17)	0.001
**pN**	pN1	257		Reference	
	pN2	25		5.96 (2.87, 12.38)	<0.001
**Extranodal Extension**	No	128		Reference	
	Yes	154		3.20 (1.39, 7.41)	0.006
**Perineural Invasion**	No	227		Reference	
	Yes	53		1.26 (0.54, 2.92)	0.59
**Lymphovascular Invasion**	No	226		Reference	
	Yes	54		2.16 (1.02, 4.60)	0.05
**Total Dose**		282		1.20 (1.04, 1.38)	0.01

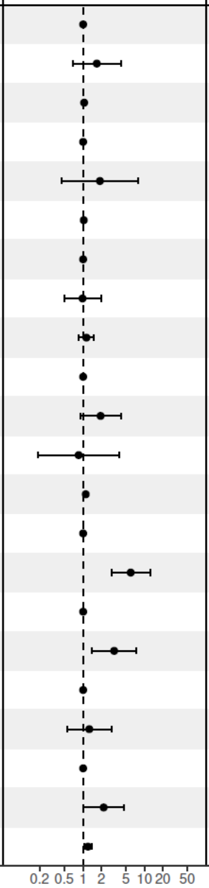

© 2026 Hidalgo CM et al. *JAMA Network Open*.

d) LRFFS

Univariable Cox Proportional Hazards Models

Variable		N	Hazard ratio		p
**Study Status**	Off Study	106		Reference	
	On Study	176		1.17 (0.30, 4.51)	0.82
**Age at Surgery**		282		1.03 (0.97, 1.10)	0.30
**Sex**	Female	30		Reference	
	Male	252		0.62 (0.14, 2.85)	0.54
**Pack Years**		278		1.04 (1.01, 1.07)	0.009
**Primary Tumor Site**	Base of Tongue	124		Reference	
	Tonsil	148		1.17 (0.37, 3.68)	0.79
**Primary Tumor Size (cm)**		269		0.97 (0.59, 1.58)	0.89
**pT**	T1	121		Reference	
	T2/T3	161		1.56 (0.47, 5.18)	0.47
**Number of Involved Nodes**		282		1.12 (1.03, 1.22)	0.007
**pN**	pN1	257		Reference	
	pN2	25		5.11 (1.54, 17.01)	0.008
**Extranodal Extension**	No	128		Reference	
	Yes	154		2.59 (0.70, 9.57)	0.15
**Perineural Invasion**	No	227		Reference	
	Yes	53		2.49 (0.73, 8.53)	0.15
**Lymphovascular Invasion**	No	226		Reference	
	Yes	54		2.49 (0.73, 8.51)	0.15
**Total Dose**		282		1.18 (0.94, 1.49)	0.16

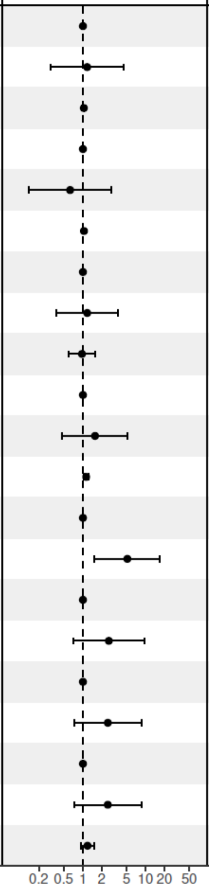

© 2026 Hidalgo CM et al. *JAMA Network Open*.
