# Supplementary material for: De-Escalated Adjuvant Radiation Therapy in Patients With HPV-Positive Oropharyngeal Cancer
Source: JAMA Netw Open. 2026 May 18;9(5):e2612837. doi: 10.1001/jamanetworkopen.2026.12837 (PMC13184782; doi:10.1001/jamanetworkopen.2026.12837)
Supplement: Supplement 2. — Data Sharing Statement [file jamanetwopen-e2612837-s002.pdf]

Data Sharing Statement

Hidalgo. De-Escalated Adjuvant Radiation Therapy in Patients With HPV-Positive Oropharyngeal Cancer. *JAMA Netw Open*. Published May 18, 2026.
doi:10.1001/jamanetworkopen.2026.12837

Data
Data available: No
